# Increasing Prevalence and Intensity of Foodborne Clonorchiasis, Hengxian County, China, 1989–2011 

**DOI:** 10.3201/eid2011.131309

**Published:** 2014-11

**Authors:** Men-Bao Qian, Ying-Dan Chen, Yi-Chao Yang, Ming-Fei Lu, Zhi-Hua Jiang, Kang Wei, Si-Liang Wei, Chang-Hai Zhou, Long-Qi Xu, Xiao-Nong Zhou

**Affiliations:** National Institute of Parasitic Diseases, Chinese Center for Disease Control and Prevention, Shanghai, China (M.-B. Qian, Y.-D. Chen, C.-H. Zhou, L.-Q. Xu, X.-N. Zhou);; Key Laboratory of Parasite and Vector Biology, Ministry of Health, Shanghai (M.-B. Qian, X.-N. Zhou);; Guangxi Center for Disease Control and Prevention, Nanning, China (Y.-C. Yang, Z.-H. Jiang);; Hengxian Center for Disease Control and Prevention, Hengxian, China (M.-F. Lu, K. Wei, S.-L. Wei)

**Keywords:** soil-transmitted helminthiases, clonorchiasis, epidemiology, prevalence, parasites, surveys, China, Clonorchis sinensis, helminths, roundworms, Ascaris lumbricoides, whipworms, Trichuris trichiura, hookworms, Ancylostoma duodenale, Necator americanus, foodborne, transmission, neglected tropical diseases

## Abstract

During 1989–2011, three parasitic disease surveys were conducted in Hengxian County, China, where soil-transmitted helminthiases and foodborne clonorchiasis are endemic. We compared the data and found that the prevalence of helminthiases decreased and the prevalence and intensity of clonorchiasis increased over time, especially among men. Clonorchiasis control/intervention measures are urgently needed in this area.

Soil-transmitted helminthiases (STHs), parasitic diseases of humans, are caused by a group of intestinal nematodes, including roundworms (*Ascaris lumbricoides)*, whipworms (*Trichuris trichiura*), and hookworms (*Ancylostoma duodenale* and *Necator americanus*) (*1*,*2*). Clonorchiasis, another parasitic disease of humans, is caused by ingestion of raw freshwater fish harboring infective *Clonorchis sinensis* metacercariae (*3*,*4*). STHs and clonorchiasis are endemic to China. STHs occur mainly in western and southern China, where environmental conditions are more favorable to helminths and social infrastructure is limited; clonorchiasis occurs predominantly in southeastern and northeastern China, where residents commonly eat raw fish. According to 2 national parasitic disease surveys conducted during 1988–1992 and 2001–2004, the prevalence of STHs in China decreased from 53.6% to 19.6% and prevalence of clonorchiasis increased from 0.3% to 0.6% between surveys (*5*,*6*).

Hengxian County (2010 population 860,000 persons), located in southern China, was one of the areas sampled in 1989 (survey 1) and 2002 (survey 2), and a third survey was conducted in the county in 2011. We used data from surveys 1–3 to determine epidemiologic changes in STHs and clonorchiasis over time in Hengxian County.

## The Study

Survey 1 sampled 1 natural village from each of 5 different towns in Hengxian County. (Towns in China have several administrative villages, the basic level of administration, which often comprise >1 natural villages) Each sampled natural village had 500–600 residents, all of whom were eligible for survey participation. The same procedures were followed in survey 2, but only 3 towns were sampled. Survey 3 sampled 8 towns and 3 administrative villages within each town. Dozens of households were surveyed from each administrative village, and all household members >5 years of age were eligible for participation; ≈200 villagers were selected in each administrative village. In each survey, a single fecal sample from each participant was used to prepare a Kato-Katz thick smear to determine the presence of helminths and count *C. sinensis* eggs.

Prevalence was determined by sex and age groups. Participants were categorized as <4 years, 5–9 years, 10–14 years, 15–19 years, 20–29 years, 30–39 years, 40–49 years, 50–59 years, 60–69 years, or >70 years of age. Overall prevalence was standardized according to data from the 2010 census for Hengxian County. We defined infection intensity as the geometric mean number of eggs per gram of feces (EPG) in the egg-positive survey participants; lg(EPG + 1) transformation was used to calculate infection intensity in all survey participants. We used Student *t* test and analysis of variance to compare infection intensities between sexes and among surveys, respectively; Fisher least significant difference test was used to compare between individual surveys. Logarithmic correlation was used to explore the relationship between prevalence and infection intensity in age groups.

Survey 1 had 2,623 participants; surveys 2 and 3 had 1,748 and 3,437, respectively (Table 1). During surveys 1–3, the standardized prevalences were 19.7%, 30.5%, and 46.5%, respectively, for clonorchiasis and 86.3%, 25.1%, and 7.0%, respectively, for STHs. The corresponding standardized prevalences were 69.8%, 13.8%, and 0.5% for roundworm infections; 55.7%, 11.2%, and 1.4% for whipworm infections; and 24.3%, 6.3%, and 5.3% for hookworm infections.

**Table 1:** Standardized prevalence of parasitic diseases, as determined from 3 surveys, Hengxian County, China, 1989–2011*

Infection	Prevalence, %										
	Survey 1, 1989				Survey 2, 2002				Survey 3, 2011		
	Female, n = 1,134†	Male, n = 1,227‡	Total, n = 2,361§		Female, n = 775¶	Male, n = 925‖	Total, n = 1,700#		Female, n = 1,641	Male, n = 1,796	Total, n = 3,437
Clonorchiasis	10.4	28.5	19.7		20.8	39.6	30.5		29.2	62.9	46.5
Soil-transmitted helminthiases**	86.2	86.3	86.3		29.5	20.9	25.1		9.4	4.7	7.0
Roundworms	70.1	69.6	69.8		16.9	10.9	13.8		0.5	0.4	0.5
Whipworms	57.0	54.5	55.7		13.1	9.4	11.2		1.7	1.0	1.4
Hookworms	27.8	21.1	24.3		7.8	4.9	6.3		7.5	3.2	5.3

*Population data were derived from the 2010 census for Hengxian County. †117 children <5 years of age were excluded. ‡145 children <5 years of age were excluded. §262 children <5 years of age were excluded. ¶24 children <5 years of age were excluded. ‖24 children <5 years of age were excluded. #48 children <5 years of age were excluded. **Some participants were infected with >1 type of helminth.

During surveys 1–3, the standardized prevalences of clonorchiasis were 10.4%, 20.8%, and 29.2%, respectively, among females and 28.5%, 39.6%, and 62.9%, respectively, among males. The corresponding standardized prevalences of STHs were 86.2%, 29.5%, and 9.4% among females and 86.3%, 20.9%, and 4.7% among males. The corresponding standardized prevalences of roundworm, whipworm, and hookworm infections among males and females showed a decreasing trend similar to that for the total STHs. 

**Figure 1:**
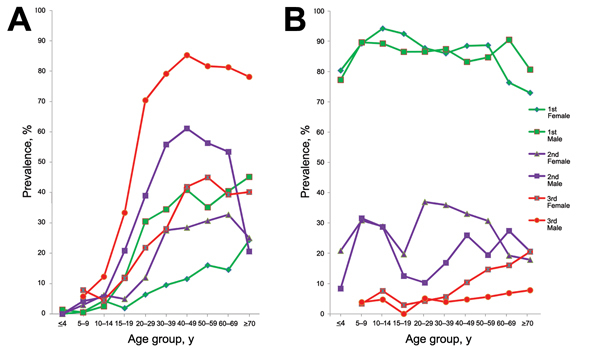
Prevalence of clonorchiasis (A) and soil-transmitted helminthiases (STHs) (B) among sex and age groups during 3 parasitic disease surveys, Hengxian County, China, 1989–2011. Green indicates the first survey (1989); purple indicates the second survey (2002); red indicates the third survey (2011).

In surveys 1–3, clonorchiasis prevalence increased fairly steadily by age group to middle age, after which, prevalence continued to increase with age in survey 1 and decreased with age in surveys 2 and 3 (Figure 1, panel A). STHs prevalence changed irregularly by age group in surveys 1 and 2 but increased gradually by age group in survey 3 (Figure 1, panel B). Prevalences of roundworm and whipworm infections usually peaked among children 5–14 years of age; hookworm infections peaked in middle-aged age groups in surveys 1 and 2 and in older age groups in survey 3.

**Figure 2:**
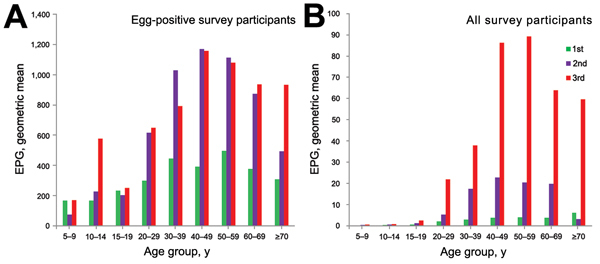
Infection intensity of clonorchiasis among persons in different age groups during 3 parasitic disease surveys, Hengxian County, China, 1989–2011. A) *Clonorchis sinensis* egg–positive survey participants. B) All survey participants. EPG, eggs per gram of feces. Green indicates the first survey (1989); purple indicates the second survey (2002); red indicates the third survey (2011).

In *C. sinensis* egg–positive participants, the geometric mean numbers of EPG were 367.3 EPG, 833.3 EPG, and 942.9 EPG in surveys 1–3, respectively (*F* = 36.2, p<0.001) (Table 2). Differences were significant between survey 1 and surveys 2 and 3 (p<0.001) but not between survey 2 and survey 3 (p = 0.210). In surveys 1–3, geometric mean numbers of EPG were 241.7 EPG, 364.9 EPG, and 405.1 EPG, respectively, among females (*F* = 3.3, p<0.05) and 419.8 EPG, 1,199.7 EPG, and 1,363.2 EPG, respectively, among males (*F* = 43.6, p<0.001). Among all participants, the geometric mean numbers of EPG were 1.6 EPG, 6.3 EPG, and 38.2 EPG during surveys 1–3, respectively (*F* = 522.9, p<0.001); differences between surveys were all significant (p<0.001). In surveys 1–3, geometric mean numbers of EPG were 0.6 EPG, 2.2 EPG, and 6.8 EPG, respectively, among females (*F* = 137.6, p<0.001) and 3.1 EPG, 13.5 EPG, and 171.5 EPG, respectively, among males (*F* = 474.4, p<0.001). Among egg-positive participants and all participants, infection intensities were all significantly lower among females than males, and infection intensities increased as ages increased, reaching a peak in middle-aged persons and then decreasing in older persons (Figure 2).

**Table 2:** Differences in infection intensity between male and female participants in 3 parasitic disease surveys, Hengxian County, China, 1989–2011*

Sex	Geometric mean no. EPG among *Clonorchis sinensis* egg–positive participants				Geometric mean no. EPG among all participants		
	Survey 1, 1989	Survey 2, 2002	Survey 3, 2011		Survey 1, 1989	Survey 2, 2002	Survey 3, 2011
Female	241.7	364.9	405.1		0.6	2.2	6.8
Male	419.8	1,199.7	1,363.2		3.1	13.5	171.5
Total	367.3	833.3	942.9		1.6	6.3	38.2

*Geometric mean number of EPG was used as the indicator of infection intensity. Children <5 years of age were excluded. EPG, eggs per gram of feces.

In surveys 1–3, clonorchiasis prevalence in different age groups was significantly correlated with the corresponding infection intensity in terms of the geometric mean number of EPG after logarithmic transformation (p<0.01). Determination coefficients (*R*^2^) were 0.709, 0.891, and 0.748 in surveys 1–3, respectively, when only egg-positive participants were included in the analysis; corresponding *R*^2^ were 0.901, 0.980, and 0.997, respectively, for all participants.

## Conclusions

Analysis of data from 3 parasitic disease surveys conducted in Hengxian County over the last 22 years showed substantial decreases in the trend of STHs prevalence and substantial increases in the patterns of clonorchiasis prevalence and infection intensity. These findings indicate a transitioning pattern of the most prominent parasitic disease changing from STHs to clonorchiasis. Distribution patterns of prevalence and infection intensity among sex and age groups showed that men have been most affected by increases in *C. sinensis* transmission and infection. Thus, control measures and health education programs are urgently needed, especially among men, to reduce *C. sinensis* disease and transmission.

National STHs surveillance showed a similar decreasing trend (*7*). This trend may partly be explained by massive health education programs and chemotherapy for STHs, which were implemented in schools in China after the first national parasitic disease survey (*8*). In addition, over the last 30 years, rapid economic development has occurred in China, resulting in increased availability of safe water and establishment of sanitary lavatories for improved hygiene (*9*). Furthermore, the increased use of chemical fertilizer has reduced the use of human feces as fertilizer (a source of helminth transmission) (*10*). However, economic development has also promoted development of aquaculture and made it economically possible for more residents to include freshwater fish in their diets, a source of *C. sinensis* metacercariae when consumed raw (*11*). 

Clonorchiasis is a neglected parasitic disease in China: no nationwide intervention or control programs have been implemented to reduce the caseload (*12*). A third national survey is expected to be conducted beginning in 2014, which will provide updated data regarding the national status of clonorchiasis and an impetus for public health control/intervention and education programs. A national clonorchiasis surveillance system should also be established to gather up-to-date information and inform public health policymakers.
